# Characteristics and Comparative Genomic Analysis of a Novel Virus, VarioGold, the First Bacteriophage of *Variovorax*

**DOI:** 10.3390/ijms232113539

**Published:** 2022-11-04

**Authors:** Przemyslaw Decewicz, Michal Kitowicz, Monika Radlinska

**Affiliations:** Department of Environmental Microbiology and Biotechnology, Institute of Microbiology, Faculty of Biology, University of Warsaw, Miecznikowa 1, 02-096 Warsaw, Poland

**Keywords:** *Variovorax*, bacteriophage, prophage, comparative genomic, Zloty Stok

## Abstract

*Variovorax* represents a widespread and ecologically significant genus of soil bacteria. Despite the ecological importance of these bacteria, our knowledge about the viruses infecting *Variovorax* spp. is quite poor. This study describes the isolation and characterization of the mitomycin-induced phage, named VarioGold. To the best of our knowledge, VarioGold represents the first characterized virus for this genus. Comparative genomic analyses suggested that VarioGold is distinct from currently known bacteriophages at both the nucleotide and protein levels; thus, it could be considered a new virus genus. In addition, another 37 prophages were distinguished in silico within the complete genomic sequences of *Variovorax* spp. that are available in public databases. The similarity networking analysis highlighted their general high diversity, which, despite clustering with previously described phages, shows their unique genetic load. Therefore, the novelty of *Variovorax* phages warrants the great enrichment of databases, which could, in turn, improve bioinformatic strategies for finding (pro)phages.

## 1. Introduction

*Variovorax* are Gram-negative, aerobic and motile bacteria belonging to the family *Comamonadaceae* of the *Betaproteobacteria* class. Members of this genus are commonly present in soil and freshwater environments and have been isolated from many locations in Europe, Asia and the Americas, as well as from polar regions [1]. Interestingly, *V. paradoxus* has also been identified as part of the methylotrophic microbiota of the human mouth [2]. The diverse distribution of these bacteria is in line with their ability to grow over a wide temperature range, as meso- and psychrophilic strains have also been discovered [3]. *Variovorax* spp. are metabolically diverse and exhibit the ability to degrade a variety of substrates [1], including a series of organic pollutants, such as BTEX (benzene, toluene, ethylbenzene and xylene), phenol, trichloroethylene (TCE), acrylamide and pesticides [4,5,6,7]. Furthermore, there has also been a report of a strain that is capable of the biotransformation of ibuprofen, using it as a sole carbon and energy source [8,9]. Another interesting feature is the ability to degrade acyl-homoserine lactones, which are signaling molecules used by many *Proteobacteria* for social communication in a phenomenon known as ‘quorum sensing’ [9].

The *Variovorax* genus is also a member of the group of plant growth-promoting rhizobacteria (PGPR), having a direct or indirect influence on the host plant; thus, it is used as model bacteria for the study of microbe–plant interactions [10]. It was reported that, in some cases, the presence of a rhizobacterium enhances the plant’s resistance to biotic and abiotic stresses [11]. Thus far, *Variovorax* spp. strains isolated from the rhizospheres of important crops, such as sunflower (*Helianthus annuus* L.) or tomato (*Solanum lycopersicum* L.), have been described [12,13]. They were also isolated from the leaf surface of the common dandelion (*Taraxacum officinale* F.H. Wigg.) and from lichen *Himantormia* [14].

Several studies have reported the extreme heavy metal tolerance of the *Variovorax* representatives that originate from metal-polluted habitats (industrialized and post-industrial areas, flotation tailing dumps, mining waste). For example, the cadmium- and cobalt-resistant 5C-2 strain was isolated from the root zone of Indian mustard (*Brassica juncea* L.) cultivated on mining waste [15,16], and RA128A was found to be resistant to multiple heavy metals, including Zn, Pb, Cd, Cu and Ag [17]. In turn, IDSBO-4 is capable of arsenite [As(III)] and antimonite [Sb(III)] oxidation [18]. The unique properties of *Variovorax* spp. make them promising candidates for research into the bioremediation of soils contaminated with heavy metal compounds [19,20].

In this work, we describe a novel *Variovorax* sp. strain, ZS18.2.2, a biofilm inhabitant in the Zloty Stok gold and arsenic mine (Poland). Unlike the strains mentioned above, which originate from the soil of the mine area or mine tailings, ZS18.2.2 was isolated from a sample of a natural microbial biofilm that covered the rock roof and walls located at the end of the two-kilometer-long Gertruda Adit in the mine [21]. The abandoned Zloty Stok mine is characterized by hash environmental conditions in terms of a stable low temperature (~10 °C), reduced concentration of oxygen (17.2%) and very high concentrations of metals, including an abundance of arsenic [21,22]. A number of arsenic-resistant strains from this site have already been characterized [23,24,25]. Interestingly, all of them were lysogens—as was the ZS18.2.2.

Bacterial viruses (bacteriophages; phages in short) are the most abundant biological entities on Earth, with an estimated 10^31^ virus-like particles in the biosphere [26]. They play a significant role in the life cycle and evolution of each bacterial genus and maintain microbial balancing in each ecosystem [27]. Most known phages have double-stranded DNA genomes packed into a tailed proteinaceous capsid. They can either be virulent or temperate [28]. Virulent phages only reproduce via the lytic cycle, while temperate phages can undergo either a lytic or a lysogenic cycle. In the latter, viral DNA persists in the host as a prophage that is integrated into the bacterial host chromosome or maintained extrachromosomally in the cytoplasm as independent episomes, replicating alongside the host DNA [29]. Prophages are a major reservoir of new genes for bacteria and can provide multiple benefits to the host—for example, in niche adaptation, biofilm formation and the production of virulence factors; they increase the host’s response to general environmental stresses and increase the host’s resistance to antibiotics and superinfection [30,31,32]. Understanding viral contributions to the evolution of bacteria and the possibility for the reshaping of bacterial cell functions by infection are some of the reasons for the renewed interest in bacteriophages. In addition, phages are commonly used to develop new genetic tools for routine genetic manipulation, such as replicative vectors and phage delivery systems [33]. Furthermore, phage-encoded proteins (e.g., depolymerases) seem to be promising tools for controlling pathogenic bacteria and other applications, including clinical diagnostics and biochemical analyses [34].

Although the number of sequenced phage genomes continues to increase, viruses infecting many ecologically important and abundant bacteria still remain poorly investigated. For example, there is a large disproportion in the number of characterized phages infecting highly studied *Gammaproteobacteria* and (pro)phages of *Betaproteobacteria* [35], which in turn hampers the understanding of the ecological roles and biological traits of the latter viruses. Among the group of understudied phages are those infecting the *Variovorax* genus. We found only one report on the lysogenicity of various soil isolates, which resulted in the identification of *Variovorax* hosts containing inducible prophages [36]; however, this mentioned survey was limited to only morphological studies of isolated viruses. In contrast to this, our study describes not only the isolation, but also contains a thorough analysis of the architecture of the *Variovorax* temperate bacteriophage genome, as well as its comparative analysis with known viruses and with putative prophage sequences that were identified by us in complete *Variovorax* genomes. Therefore, this is the first report of an active *Variovorax* phage that includes its complete genome sequence and characterization. These findings will help to fill the gap in our understanding of previously unrecognized *Variovorax* phages and substantially expand our current knowledge regarding the genomic diversity of bacterial viruses.

## 2. Results and Discussion

### 2.1. Identification and Characterization of Variovorax sp. strain ZS18.2.2

A new *Variovorax* sp. strain, designated ZS18.2.2, was isolated from a sample of rock biofilms from the Zloty Stok gold and arsenic mine—collected in February 2021. When grown on solid R2A medium, this strain formed colonies with mucoid morphology (Figure S1), possibly due to its EPS-producing ability, which suggests that the strain is actively involved in biofilm production; moreover, surface motility, similar to swarming motion, has also been observed at 4, 10 and 20 °C on semi-solid (0.5%) R2A and LB media, and at 30 °C only on R2A. Similar properties were reported and examined for the reference strain, *V. paradoxus* EPS [13]. To determine the temperature requirements of *Variovorax* sp. ZS18.2.2 for growth, we performed a temperature tolerance analysis on plates with LB and R2A media. This test revealed that ZS18.2.2 grew in both media at temperatures ranging between 4 °C and 30 °C (but not at 37 °C). Therefore, this bacterium can be considered psychrotolerant and, consequently, well adapted to living at the stable low temperatures of the Zloty Stok gold mine.

As ZS18.2.2 was isolated from an arsenic-rich environment where the concentration of arsenic hydride reached 1.52–3.23 mg/m^3^ [21], the resistance of this strain to inorganic arsenic species was tested. ZS18.2.2 showed extreme tolerance to As(V) (up to 300 mM) and moderate resistance to As(III) (up to 10 mM). Therefore, *Variovorax* sp. ZS18.2.2 is another strain isolated from the Zloty Stok mine that has shown considerable arsenic resistance; moreover, when ZS18.2.2 grows in R2A medium containing 5 or 10 mM of As(III), it oxidizes As(III) to As(V). In comparison to other known *Variovorax* strains that are resistant to As(III) or As(V), ZS18.2.2 was found to be much more tolerant to As(V) than e.g., *Variovorax* sp. MM-1 (200 mM) [37], but less tolerant to As(III) than *V. paradoxus* Dhal F [38] and *Variovorax* sp. MM-1 [37], which showed resistance up to 15 mM and 20 mM, respectively. To the best of our knowledge, *Variovorax* sp. ZS18.2.2 shows the highest resistance to As(V) compounds among all *Variovorax* strains that have been isolated and tested for this characteristic so far (Table S1).

The total DNA of ZS18.2.2 was isolated and sequenced. The reconstruction of its genome resulted in 46 contigs with a total length of 7,235,762 bp and 66.32% average GC content. A putative extrachromosomal element—plasmid (112,571 bp)—was found (JANLNM010000015).

We searched the ZS18.2.2 genome for putative prophage sequences using the PhiSpy program [39]. The analysis revealed the presence of such a region, within contig 3 (JANLNM010000003). To verify whether the recognized prophage region was an active virus, we treated the ZS18.2.2 cells with mitomycin C, a classical inducer of lambdoid prophages. This approach caused the induction of the phage named VarioGold. Its DNA was isolated from capsids and re-sequenced. The obtained reads confirmed the previously recovered prophage sequence and also allowed for the determination of phage termini (see below).

### 2.2. Morphological Analysis by TEM

**Figure 1:**
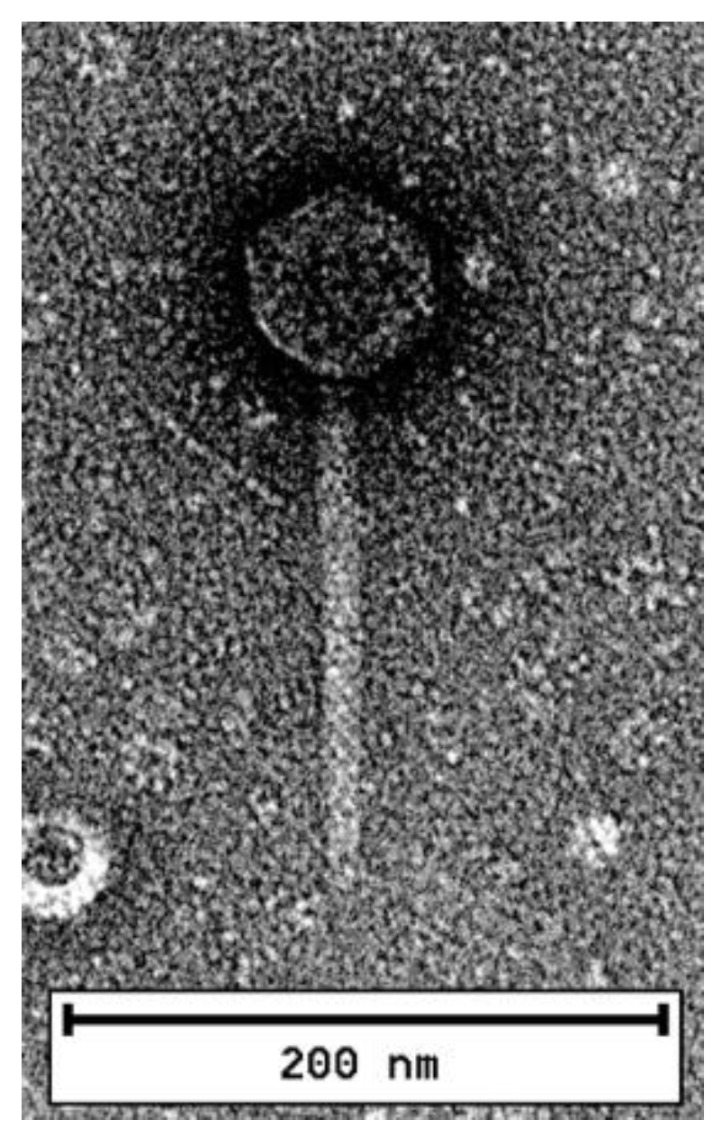
Visualization of the VarioGold virion.

The VarioGold phage was subjected to TEM analysis to determine its morphotype. Its virions had an icosahedral head (60 nm in diameter) and a flexible, non-contractile tail (approximately 170 nm long), thus showing a siphovirus morphotype (Figure 1).

### 2.3. Host Range Testing

The ZS18.2.2 strain was the only member of the *Variovorax* genus isolated from the Zloty Stok gold mine. Therefore, we used two model strains of *V. paradoxus* EPS and B4 as potential hosts for VarioGold in the spot test. None of the tested strains supported the detectable lytic growth of VarioGold. Presumably, VarioGold is highly specific and has a narrow host range, possibly confined to its natural host strain, ZS18.2.2—although this hypothesis needs experimental validation with the use of a larger number of *Variovorax* strains.

### 2.4. Genomic Analysis of the VarioGold Phage

#### 2.4.1. Identification of the Genome Termini of the VarioGold Phage

The genome of the VarioGold phage consists of 39,429 bp linear double-stranded DNA molecules with a GC content of 62.7%, which is somewhat lower than that of the host (66.32%; see above). The VarioGold genome termini and its possible packaging strategy were analyzed using the PhageTerm tool [40]. This program predicted that VarioGold had linear genomic DNA with fixed termini, i.e., 10 nt 3′ cohesive ends, and that the protruding overhang had a nucleotide sequence of CGATCGGTTC. This suggests that VarioGold utilizes the cohesive-end packaging strategy to package its genome. As cohesive ends are covalently joined together in the prophage state, forming a so-called cos site, we compared the VarioGold prophage sequence within contig 3 of the ZS18.2.2 draft genome with those of the corresponding regions of the DNA isolated from free phage particles. This alignment revealed that the VarioGold prophage indeed contains one 10 bp cos site.

#### 2.4.2. Identification of the VarioGold Attachment Site

Most integrases of prokaryotic viruses use the tRNA and tmRNA genes of its host as integration sites [41]. We discovered that the VarioGold prophage is flanked by duplicates of the 15 bp sequence (CCTCCCTCTCCTCCA), which most likely constitutes the bacterial attachment site (attB). One of its copies forms the 3′ part of a tRNA-Ser (CGA)-encoding gene that is located 176 nucleotides upstream of the 5′ end of the putative integrase gene (*VG_p29*). There is only one copy of this 15 bp sequence in the VarioGold genomic DNA that was isolated from capsids.

#### 2.4.3. Module Analysis of the VarioGold Genome

The VarioGold genome was predicted to contain 52 open reading frames (ORFs), with 33 genes (63%) located on the sense strand and 19 genes (37%) on the antisense strand. A function was assigned to 38 ORFs (73% of the total gene number). The remaining 14 ORF-encoded proteins showed similarities with hypothetical proteins that were already described but were of an unknown function. No gene-encoding tRNA was detected in the genome. The positions, sizes and putative functions of the proteins are listed in Table 1.

**Figure 2:**
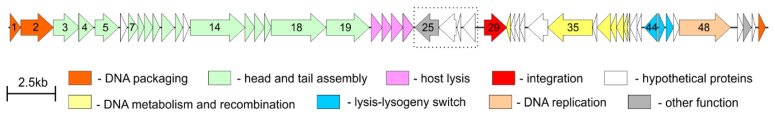
Genome map and functional annotation of the predicted ORFs of the VarioGold phage. Each arrow represents an ORF and its direction corresponds to the direction of gene transcription. The numbers on arrows refer to the ORF number in the genome, e.g., 1—*VG_p01*. The white color of arrows refers to genes encoding proteins with unknown function. The dotted line indicates a cluster of genes (including *VG_P25* encoding O-antigen ligase) that are predicted to be jointly regulated and expressed in the VarioGold prophage (putative moron-like genes).

VarioGold displays a modular genome that can be divided into five functional modules: DNA packaging; virion structure and morphogenesis; host lysis; integration–excision; lysis–lysogeny switch; and genome replication (Figure 2).

In all members of the *Caudoviricetes* class, a complex of two proteins commonly referred to as terminases accomplish DNA encapsidation when preformed empty procapsids are subsequently filled with the viral genome by means of a DNA packaging machine [42]. The protein products of the *VG_p01* and *VG_p02* genes that are probably involved in the VarioGold genome packaging showed the highest sequence similarity to their counterparts encoded by myovirus *Burkholderia* BgVeeders33 (UEW68542.1, UEW68541.1. 31% and 54% amino acid identity, respectively) and siphovirus *Marinobacter* AS1 (MK088078.1, MK088078.1, 29% and 43% identity, respectively). In many sequenced tail phage genomes, a gene-encoding HNH endonuclease is located next to their cohesive end site and terminase genes, suggesting a role of HNH proteins in the endonuclease and/or packaging activities of the terminases [43]. Therefore, the position in the VarioGold prophage of the *VG_p52* gene (whose protein product contains the putative HNH endonuclease domain, PF01844)—immediately adjacent to the *VG_p01* and *VG_p02* genes—suggests its potential involvement in DNA packaging. Interestingly, HNH endonucleases encoded by genes adjacent to the terminases of the abovementioned BgVeeders33 and AS1 phages showed similarity to VG_p52 (38 and 35% identity, respectively).

Eighteen ORFs located downstream of the packaging module may have assigned functions based on their homology to structural proteins encoded by other phages: portal (p03), scaffold (p04), major head (p05), head–tail connector (p08), head closure tail component (p09) and tail (p10-p19) proteins. In the case of VG_p06, no viral counterpart has been found. This protein had little similarity with a few hypothetical *Betaproteobacteria* proteins. Interestingly, the HHpred search for a putative tail spike of VarioGold (VG_p19) identified a hit (with 100% probability, E value, 3.4 × 10^−45^) with pectate lyase superfamily proteins. These types of virion-associated enzymes are used by phages for the degradation of bacterial capsular polysaccharides and are called depolymerases [34,44].

The virion structural module in the VarioGold genome is followed by a cluster of five genes (*VGp20*-*p24*) that were predicted to comprise the lysis module. The VG_p22 protein contains a lysozyme-like motif (pfam00959); thus, it presumably acts as an endolysin (murein hydrolase) that cleaves the β-1,4-linkages between adjacent N-acetylmuramic acid and N-acetylglucosamine residues in cell wall peptidoglycans. The gene located upstream of *VG_p22* encodes a putative holin (VG_p21) and was predicted to contain three transmembrane helices and an N-terminal signal peptide. VG_p23 and VG_p24 are probably the inner and outer membrane subunits of the spanin complex; they have a single N-terminal transmembrane domain and a lipoprotein outer membrane localization signal, respectively. A BLASTP search in the NCBI virus database revealed a homolog for VG_p22 only (i.e., YP_009100017.1 of *Escherichia* phage vB_EcoM-ep3, 39% identity).

The VG_p29 gene is predicted to encode an integrase, as its protein product belongs to the tyrosine recombinase family (pfamPF00589). Its most closely related viral proteins are tyrosine integrases from *Rhodobacter* siphophages, e.g., RcPutin (GenBank accession QXN72045.1), and RcRios (QXN72145.1), which share 39% identity with the VG_p29 protein. The putative VarioGold integrase gene is located in the opposite orientation to the segment of 15 mostly hypothetical genes (unassigned function). The functional domains were detected in VG_p35 (ParB-like nuclease domain, pfam02195), VG_p37 (Nucleoside/nucleotide kinase superfamily, cl17190), VG_p38 (P-loop ATPase family, cl38936) and VG_p39, which was predicted as a putative single-stranded DNA-binding protein. 

The last gene (towards the 3′ genome end) of this segment is *VG_p44*, which, together with the oppositely oriented *VG_p45*, comprises a putative lysis–lysogeny locus that is responsible for switching between the lytic and the lysogenic cycles. The carboxyl terminal region of VG_p44 contains the S24 signal peptidase domain (pfam00717) that is typical of CI repressor-like proteins. Its homologs were found among protein products encoded in several assembled genomes of uncultured viruses and *Burkholderia* phage vB_BmuP_KL4 (YP_009800704.1, 35% identity). The putative transcriptional regulators, Cro-like VG_p45 and CII-like VG_p46 (PF06892), had no homologs in the NCBI viral database but showed similarity with those encoded in bacterial genomes.

Two domains were identified in VG_p48, including Toprim (PF13362; from 227 to 302), which is found in bacterial DnaG-type primases and their viral counterparts [45], and virulence-associated protein E (PF05272; from 574 to 755). While the segment of VGp_48 spanning the latter domain showed similarity to several putative phage primases (e.g., *Marinobacter* phage PS3, ATN93361.1 [46]), VG_p48 was homologous over its entire length only with the protein (DAS38011.1) encoded by a virus assembled from human metagenomes [47]. 

A putative protein product of the *VG_p50* gene showed no viral homologs, but the HHpred search identified a hit for antiterminator Q-like proteins (PF06530) that modify the RNA polymerase near the phage late-gene promoters and thereby cause antitermination at distant sites [48]. 

The most intriguing gene of VarioGold is *VG_p25*, whose protein product shows a 31% amino acid identity with the O-antigen ligases encoded by *Pseudomonas* phages D3 (NP_061525.2) [49], phi297 (YP_005098089.1) [50] and PP9W (UAW06749.1). A conserved Wzy_C superfamily motif (also called an O-Ag ligase domain due to its prevalence in WaaL proteins found in various Gram-negative species that catalyze a key step in lipopolysaccharide synthesis [51]) was identified within the C-terminal half of the VG_p25 protein (residues 200–326); moreover, the TMPred and TMHMM programs predicted ten strong transmembrane regions in VG_p25. The presence of multiple transmembrane helices is a characteristic feature of the O-Ag ligase family proteins [52]. It was revealed that the D3 phage causes a serotyping switch of *P. aeruginosa* serotype O5 to O16; thus, during lysogeny, the D3-modified LPS receptors of its host are resistant to LPS-dependent phages. Three proteins of the D3 phage are responsible for this seroconversion phenomenon: an O-acetylase (Oac, NP_061524.1), the abovementioned O-Ag ligase (NP_061525.2), and an α-polymerase inhibitor (Iap, YP_009173780.1). The genes that encode these proteins are not organized as an operon, as *iap* and O-Ag ligase are located on the complementary strand to *oac*, which is located on the top strand between these genes [53]. The *VG_p25* gene is adjacent to the oppositely oriented host cell lysis gene cluster (*VG_p20-p24*; see above) and to the similarly oriented three hypothetical genes (*VG_p26-p28*). The common feature of protein products of the latter is the presence of transmembrane helices and/or an N-terminal signal sequence; however, none of these VarioGold putative proteins are similar to the Iap and Oac encoded by the D3 phage. Although it is highly speculative, we suppose that the putative O-antigen ligase gene of VarioGold and the other upstream-located genes (*VG_p26-p28*) form a common, collectively regulated cluster because they are all oriented in the same direction, unlike the late genes of this phage (that is, those of replication, structural and host lysis modules); if so, perhaps they are active in lysogeny.

### 2.5. Comparative Genomic Analyses

#### 2.5.1. Comparative Analysis of VarioGold with Other Phages 

**Figure 3:**
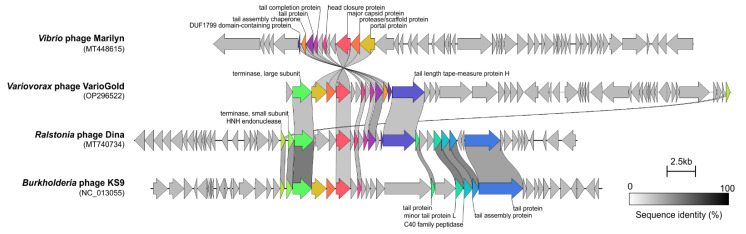
Comparative genome alignment of VarioGold and the three phages. The alignment was created with Clinker using default settings. Each ORF is represented by an arrow. ORF-encoding proteins that did not share sequence similarity are colored gray, while others are connected with blocks reflecting the degree of sequence identity and are color-coded. Sequence identity ranged between 32% and 60%.

A nucleotide BLAST search of the NCBI viral genome database using the complete genome sequence of VarioGold did not reveal any significant matches covering more than 3% of the query genome. Therefore, to determine the location of VarioGold within the phage population network, a viral cluster analysis with vConTACT2 [54] was conducted, and the ViPTree whole-genome-based phylogenomic tree [55] was constructed. The network indicated that VarioGold was an outlier, and it was exclusively connected via edges with three temperate siphoviruses—two infecting members of *Alphaproteobacteria* (genera: *Ralstonia* and *Burkholderia*) and one infecting the *Vibrio* genus of *Gammaproteobacteria*. The Clinker alignment showed that VarioGold shared eight, seven or six similar DNA packaging and structural proteins with them (Figure 3); these were: (i) *Vibrio* phage Marilyn (portal, protease, major capsid, head closure, tail completion protein, tail protein, tail assembly chaperone, DUF1799 domain-containing phage protein); (ii) *Ralstonia* phage Dina (HNH, TerL, major capsid, head closure, tail completion, tail and tape measure proteins); and (iii) *Burkholderia* phage KS9 (HNH, TerL, portal, protease/scaffold, major capsid, head closure). Among these, the KS9 and Dina phages were also the ones with which VarioGold created a clade in the ViPTree analysis based on the limited similarity between regions encoding the mentioned proteins (Figure S2). These three phages have not been completely taxonomically classified. Marilyn (MT448615) and KS9 (FJ982340) are without not only genus-level but also family-level taxonomy information, while phage Dina (MT740734) was classified as the only representative of the Dinavirus genus [56]; moreover, the VarioGold genome does not share any nucleotide sequence similarity with the Dina, Marilyn and KS9 genomes based on both BLASTN searches and OrthoANIu [57] analyses. In the case of the latter, the only similarity was observed between VarioGold and Dina phages across 669 bp with 58.5% OrthoANIu values. These results suggest the lack of a significant phylogenetic relationship between VarioGold and known phages, thus indicating that VarioGold is a novel virus; thus, regarding current taxonomic assignment procedures, it may be considered a new viral genus [54,58,59].

#### 2.5.2. Identification and General Genomic Features of Variovorax spp. Prophages

Since VarioGold is the first bacteriophage of *Variovorax* spp., no comparisons could be made to other *Variovorax* phages; therefore, we attempted to identify prophage sequences in complete *Variovorax* spp. genomes that were extracted from the NCBI database.

As of 25 October 2021, 21 publicly available complete genomes of the *Variovorax* genus (Table S2) were available and these were searched for the presence of putative prophages, as previously reported [60,61]. Briefly, for the initial integrase and attL/attR prediction, the PhiSpy algorithm was used [39] and manually inspected by assessing the phage localization in the host genome. Subsequently, we verified the presence of the essential structure and packaging genes using Virfam (http://biodev.cea.fr/virfam/, accessed on 17 January 2022) and BLASTP [62]. Only the regions containing complete prophage genomes were included in further analyses. All prophage elements lacking matches to core phage proteins (e.g., terminase, capsid, head, tail proteins) were excluded. Genome annotation was further verified using the MAISEN web service in order to determine the genomic context of the investigated genes [63].

As a result, 37 novel prophages were identified (Table 2). These were detected in almost all *Variovorax* spp., except *Variovorax* sp. PAMC-28562, PBL-H6 and SRS16. Among them, 10 out of 21 were found to be polylysogenic, carrying multiple prophages within their genomes: five strains had more than two complete prophage regions, of which two strains (*Variovorax* sp. PMC12 and *V. paradoxus* VAI-C) had four such sequences, and a single strain (*V. boronicumulans* J1) had five. Virfam analysis indicated that, presumably, there were 17 of siphoviral morphology, 16 of podoviral morphology and 4 of myoviral morphology. The integration modules of the identified prophages encoded tyrosine recombinases (33 prophages), serine recombinases (3) or Mu-like transposases (2) (Table 2).

Putative integration sites were identified for the majority of the tyrosine recombinase-encoding *Variovorax* viruses (Table 2). For 28 prophages, these sites were various tRNA genes, of which the most commonly targeted were (i) tRNA_Arg_, which was used by seven prophages, and (ii) tRNA_Ser_, each of which were used by nine prophages and also by VarioGold. These observations corroborate previous findings regarding the preferential integration of phages (and other integrative elements) within tRNA genes [64].

The genome size of the identified prophages ranged from 38 kb to 113 kb. Total prophage genomes accounted for 0.5–6% of the bacterial chromosome, which appeared to be lower compared to that of other bacterial genomes (10–20%). As examples, the genomes of the *Streptococcus pyogenes* and *Escherichia coli* O157:H7 strains contain 12% and 16% prophage sequences, respectively [65]. The genomes of identified prophages were aligned with Clinker to explore the similarities in their structures and encoded proteins (Figure S3). The analysis revealed high mosaicism of their genomes and sets of unique proteins encoded by each prophage. The most distinctive ones were 5C-2_pp_1, HW608_pp_2, PBS-H4_pp_1 and PBS-H4_pp_2, which shared—at most—seven proteins (based on at least 30% sequence identity) with other prophages. Despite that, eight groups of prophages encoding more similar proteins could be indicated.

**Figure 4:**
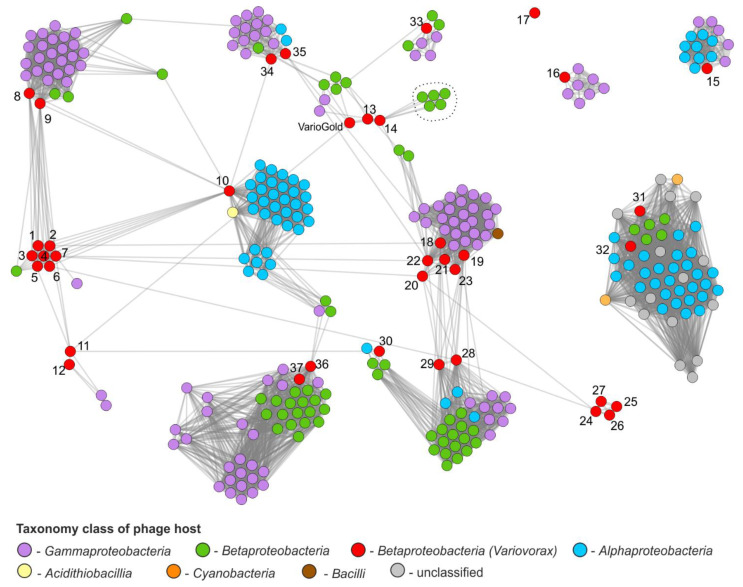
Proteome-based similarity network. The network was constructed with vConTACT2 to explore the diversity of *Variovorax* prophages in comparison with other known phages. Each node represents a single phage genome, and the edge represents a significant similarity between proteomes of connected phages. Dashed lines surrounding five phages indicate *Inoviridae* phages RSS0, RSS1, RSS-TH1, RS611 and RSBg infecting *Ralstonia* spp. Numbers next to *Variovorax* prophages correspond to numbering in Table 2.

To better understand the diversity and relationships among prophages of *Variovorax* spp., protein-sharing networks were generated with vConTACT2 and the INPHARED database (1 December 2021 release, over 17,470 phage genomes). In the resulting network, *Variovorax* prophages demonstrated significant proteome similarity to, in total, 280 known phages (Figure 4 and Figure S4). Prophages were spread across various clusters, except for one—HW608_pp_2—which remained a singleton. They were often located outside clusters and acted as bridges between them, thereby reflecting their mosaic genome structure. Interestingly, the prophages present in the same strain (J1_pp1 and J1_pp4; J1_pp2 and J1_pp3; PMC12_pp3 and PMC12_pp4) are similar to each other and clustered together, which is not common.

According to vConTACT2, 7 *Variovorax* prophages were considered to be outliers (PBS-H4_pp_1, PBS-H4_pp_2, HW608_pp_2, HW608_pp_3, 5C-2_pp_1, VAI-C_pp4, J1_pp5) and 3 were considered clustered/singletons (PAMC28711_pp2, PMC12_pp1, VAI-C_pp2), while 18 form 5 *Variovorax*-specific viral clusters (including the one with VarioGold; see below), 5 form extended viral clusters with known viruses (PMC12_pp_3 and PDNC026_pp_1; WDL1_pp_2 and PAMC26660_pp_3; PDNC026_pp_2), and others overlapped multiple viral clusters. 

The highest similarities were found for seven siphoviral prophages, i.e., PMC12_pp_2, VAI-C_pp_1, B4_pp_1, CSUSB_pp_1, vvax_pp_1, J1_pp_1 and J1_pp_4, which cluster within the same clique (numbers 1–7 in Figure 4). They also shared similarities in several tail proteins with almost identical Mu-like prophages PAMC 26660_pp_1 and RKNM96_pp_1, which were also predicted by Virfam as siphoviruses (numbers 8–9 in Figure 4). All of them were connected with siphovirus HW608_pp3 (number 10 in Figure 4).

Another group is formed by four prophages with predicted podoviral morphology, PAMC 28711 _pp_1, RA8_pp_1, PBL-E5_pp_1 and WDL1_pp_1 (numbers 24–27 in Figure 4), which shared, among other characteristics, a similar integration site in the tRNA_Arg_ gene (Table 2).

It is also worth noting that prophages PMC12 _pp_1 and RKNM96_pp_2 (numbers 31–32 in Figure 4) are a part of a dense clique that gathers representatives of the *Autographiviridae* family and are characterized by encoding their own single subunit RNA polymerase (RNAP) and a common unidirectional gene arrangement. Both these features are shared with this family by the PMC12 _pp_1 and RKNM96_pp_2 prophages. The RNAP (AVQ80729.1) of PMC12_pp_1 showed a 56% identity with its counterparts of virulent *Ralstonia* phages (e.g., P-PSG-11, [66]) and *Bordetella* phage vB_BbrP_BB8 (QDB70995.1). The putative RNAP of RKNM96_pp_2 shares a 36% identity with the RNAP encoded by *Rhizobium* phages vB_RleA_TRX32-1 and RHEph01 [67] and also the RNAP of the temperate *Teseptimavirus* S2B-infecting *Caulobacter crescentus* CB15 [68]. Until recently, T7-like phages were considered strictly virulent. Nevertheless, several putative representatives of the *Autographiviridae* family have been identified lately in several Gram-negative and several Gram-positive bacterial genomes, or ones that are able to establish a lysogenic relationship with their host—for example, *Teseptimavirus* S2B, which is mentioned above. Therefore, prophages PMC12_pp_1 and RKNM96_pp_2 would be other examples of these.

A somewhat surprising result of the vConTACT2 network analysis is the presence of a group of single-stranded *Inoviridae* phages that share three proteins with the J1_pp3 prophage, which were predicted to be a zonular occludens toxin, minor coat protein and attachment protein (ATA54428.1, ATA54427.1, ATA54426.1, respectively); the consecutive genes that encode them form a segment that interrupts the structural module of the J1_pp3 prophage.

VarioGold showed the highest similarity with prophages J1_pp2, VAI-C_pp2 and J1_pp3, sharing 19, 8 and 7 encoded proteins with them, respectively (Figure S5); moreover, 17 protein products of VarioGold genes could be considered unique as they did not show any similarities with proteins encoded by the *Variovorax* prophages identified by us. These are proteins encoded by putative early genes (VG_p30-34 and VG_p40-41), a lysogenic switch module (CI, Cro, CII), TerS, structural proteins (VG_p06-07), the host recognition–lysis module (tail–spike, lysozyme) and the moron-like segment of the right end of the VarioGold prophage sequence (VG_p25-p28; see above). 

## 3. Materials and Methods

### 3.1. Bacterial Strains, Media and Growth Conditions

For bacterial isolation, rock biofilm samples of the Zloty Stok gold and arsenic mine (SW Poland), which were collected in February 2021, were mixed vigorously in 0.7% NaCl and then serially diluted in saline. Aliquots of 100 µL of each dilution were plated on Reasoner’s 2A (R2A) medium, solidified with 1.5% (*w/v*) agar [69] and incubated at 17 °C for 7 days, followed by incubation at 6 °C for a further two weeks. The obtained colonies were used to isolate pure cultures. *Variovorax paradoxus* EPS [13] and *Variovorax paradoxus* B4 [70], used in host range testing, were routinely grown under aerobic conditions in R2A medium at 20 °C.

For the temperature tolerance analysis, *Variovorax* sp. ZS18.2.2 was plated with the use of the streak plate technique. The plates were incubated for 7 days at various temperatures ranging from 4–37 °C and examined after every 24 h. The data were obtained from three independent experiments.

### 3.2. Determination of the Minimum Inhibitory Concentrations of Arsenite and Arsenate

The minimum inhibitory concentrations (MIC) of arsenite and arsenate were established using the broth dilution method. Sterile tubes (15 mL) containing R2A medium amended with the respective amount of arsenite/arsenate were inoculated with overnight cultures to a final optical density at 600 nm (OD_600_) of 0.045 and incubated for 48 h at 20 °C with shaking (150 RPM). The optical density was measured immediately after inoculation and every 24 h with an automated plate reader (Sunrise, TECAN, Männedorf, Switzerland). The following arsenic compounds were used for MIC determination: NaAsO_2_ (0–50 mM) and Na_2_HAsO_4_ (0–500 mM). The MIC was defined as the lowest concentration of As^n+^ that completely inhibited the growth of bacteria. The data were obtained from three independent experiments.

### 3.3. Oxidation and Reduction of Arsenic Compounds and Arsenic Speciation Assay

In order to investigate the strain’s potential for arsenic oxidation and reduction, aerobic cultures in the R2A medium amended with the respective amount of arsenite/arsenate were set as previously performed in the MIC experiment (see Section 3.2) and incubated for 48 h at 20 °C with shaking (150 RPM). Arsenic species present in culture supernatant after 48 h of incubation were determined by the silver nitrate test described by Drewniak et al. [23]. Supernatant from each culture (200 μL) was taken and mixed at a ratio of 1:20 with a 50 mM silver nitrate (V) solution. The addition of AgNO_3_ solution to the culture containing As(III) compounds caused the precipitation of a yellow Ag_3_AsO_3_ (silver arsenite). The presence of As(V) compounds caused the precipitation of a brown Ag_3_AsO_4_ (silver arsenate).

### 3.4. Standard Molecular Biology Procedures

Standard DNA manipulations were carried out according to the protocols described by Sambrook and Russell [69].

### 3.5. Induction and Isolation of Phage Particles

To induce a potential prophage in *Variovorax* sp. ZS18.2.2, bacterial cells were treated with mitomycin C (500 ng/mL, MilliporeSigma, Darmstadt, Germany), and their growth (with shaking) was continued for 18 h. The resulting lysate was cleared of cell debris by centrifugation (13,000 RPM, 30 min). The supernatant was condensed on an Amicon ultrafiltration column Ultracel-100K (Merck Millipore, Ireland) and used for further analysis. 

### 3.6. DNA Isolation and Sequencing and Bioinformatics

The total DNA was isolated from *Variovorax* sp. ZS18.2.2 using a Genomic Mini Kit (A&A Biotechnology, Gdansk, Poland). The whole-genome shotgun sequencing of the ZS18.2.2 strain was conducted by Eurofins Genomics Germany GmbH (Ebersberg, Germany) on an Illumina NovaSeq6000 platform at a read length of 2 × 150 bp.

DNA of the VarioGold phage was isolated by phenol–chloroform extraction and isopropanol precipitation [69], and its sequencing was also performed by Eurofins Genomics with the same parameters as for bacterial genomic DNA.

The raw reads acquired from the above sequencing projects were subjected to a quality check and filtering with the application of FastQC v.0.11.5 [71] and fastp v.0.21.0 [72]. During the fastp run, the following parameters were applied: --detect_adapter_for_pe --cut_window_size 8 --cut_tail --cut_mean_quality 24 --length_required 50 --length_limit 160 --n_base_limit 5 --trim_poly_x --poly_x_min_len 0 --correction --overlap_len_require 20 --overlap_diff_limit 5. The filtered reads were then assembled with SPAdes v.3.15.3 in an isolated mode with the following kmers: 33, 55, 77, 99 and 127 [73]. The analysis of the genomes’ sequence coverage, including the analysis of the redundant regions, was performed by mapping the filtered reads against the assemblies with bwa mem v.0.7.17-r1198-dirty [74] and samtools v.1.10 [75]. Then, the alignments were viewed in Integrative Genome Viewer v.2.6.2 [76]. Additional analysis of the phage termini and packaging mechanisms was conducted with the application of PhageTerm v.1.0.12, using the filtered reads as the input [40].

### 3.7. Transmission Electron Microscopy (TEM) 

TEM analysis was conducted as described previously [77]. The visualization of the phages was performed at the Core Facility of the International Institute of Molecular and Cell Biology (IIMCB, Warsaw, Poland).

### 3.8. Genome Annotation

The analysis of the nucleotide sequence of the VarioGold phage was performed using Clone Manager 8 (Sci-Ed) and Artemis v18.0.0 software [78]. The genomes were automatically annotated using the RASTtk [79] in phage mode on the PATRIC website [80]. The annotations were manually verified based on homology searches using BLAST programs against the NCBI non-redundant (nr) and SwissProt databases [62], HHpred using HHpred or HMMER tools against the PDB_mmCIF70_11_Oct, SCOPe70_2.07, COG_KOG_v.1.0 and Pfam-A_v35; and NCBI_Conserved_Domains (CD)_v3.18 [81], InterProScan v5.48 [82], Pfam [83] and UniProt [84]. The transmembrane helices were identified with the help of the TMHMM v2.0 server [85]. Putative tRNA genes were identified using the tRNAScan-SE [86] and ARAGORN programs [87]. A phage morphotype search was carried out using Virfam [88]. Phage termini were predicted using PhageTerm [40]. All software was run at default settings.

### 3.9. Comparative Genomics Analysis

Comparative genomics analysis of the phage genomes was performed with the application of Clinker using the default settings [89]. If necessary, the genomes were circulated and re-oriented to enhance the overview of genome structure conservation. The genome of the VarioGold phage was also compared with other known phage genomes recovered from the INPHARED database (as of the 1 December 2021 release) [90] with the application of vConTACT2 v0.9.20, as described previously [54]. All of the analyzed networks were visualized with Gephi v.0.9.2 [91], and the nodes were laid out in two-dimensional space with the application of the ForceAtlas 2 [92] and Noverlap algorithms.

For phylogenetic analysis, the VarioGold phage sequence was uploaded to the ViPTree server updated on 20 June 2022 [55]. The analysis was run against all dsDNA prokaryotic viruses with automatic gene prediction. Then, the resulting tree was recalculated with a subset of neighbor phages. Selected genomes were compared with VarioGold using BLASTN (with e-value lower than 0.1 threshold) and OrthoANIu [57] to determine its taxonomic membership.

### 3.10. Nucleotide Sequence Accession Numbers

The draft genome of *Variovorax* sp. ZS18.2.2, as well as the VarioGold phage genome, can be accessed under the following GenBank accession Nos: JANLNM000000000 and OP296522, respectively.

## 4. Conclusions

In this study, we identified and characterized the first *Variovorax* virus—an inducible temperate phage, VarioGold—residing in the *Variovorax* sp. strain ZS18.2.2’s genome, which was isolated from a biofilm collected from the Zloty Stok gold and arsenic mine (Poland). The slight resemblance of VarioGold to other known phages at both the nucleotide and protein levels suggests that it should be considered a new viral genus of the *Caudoviricetes* class. We performed an insightful analysis of 21 publicly available *Variovorax* complete genomes, and in 18 of them, an additional 37 complete prophage sequences were identified. It is striking that almost all of the analyzed *Variovorax* genomes carry at least one prophage, and as many as ten of them carry more than one, which allows us to conclude that polylysogeny is common in the *Variovorax* genus; moreover, a protein-based similarity network showed the high diversity of these phages. Finally, a global analysis of the identified (pro)phages with known viruses revealed that they show a diversified level of similarity to them, often acting as bridges in network analyses, which revealed that they are partly similar to the linked groups of highly similar phages, thereby filling the gaps of viral dark matter. This work significantly expands current knowledge on the diversity of bacteriophages that infect *Betaproteobacteria*; moreover, it provides great evidence regarding how much information about the viral ‘dark matter’ can be obtained by conducting simple and low-cost analyses of bacterial genomic sequences that are deposited in public databases.

## Figures and Tables

**Table 1:** Genes located within the VarioGold phage.

ORF	Coding Region (bp)	Strand	Protein Size (aa)	Predicted Function
1	99..653	+	184	Terminase small subunit
2	653..2350	+	565	Terminase large subunit
3	2347..3702	+	451	Portal protein
4	3668..4429	+	253	Protease/scaffold protein
5	4506..5735	+	409	Major capsid protein
6	5821..6168	+	115	Hypothetical protein
7	6245..6736	+	163	Head-tail connector protein
8	6733..7059	+	108	Head closure protein
9	7067..7516	+	149	Tail-component
10	7513..7866	+	117	Tail completion protein
11	7974..8618	+	214	Tail protein
12	8677..9057	+	126	Tail assembly chaperone
13	9084..9395	+	103	DUF1799 domain-containing phage protein
14	9446..12,292	+	948	Tail length tape-measure protein H
15	12,292..12,783	+	163	Minor tail protein
16	12,783..13,289	+	168	Minor tail protein
17	13,286..13,654	+	122	NlpC/P60 family protein
18	13,651..16,491	+	946	Tip attachment protein J
19	16,511..18,742	+	743	Tail spike-like protein
20	18,841..19,407	+	188	4 TM segments containing protein
21	19,391..19,888	+	165	3 TM segments containing protein; holin
22	19,885..20,490	+	201	Phage lysozyme
23	20,487..21,035	+	182	Spanin, inner membrane subunit
24	20,731..20,997	+	88	Spanin, outer lipoprotein subunit
25	21,090..22,325	−	411	10 TM segments containing protein; O-antigen ligase family protein
26	22,340..23,239	−	299	Hypothetical protein; N-term signal peptide
27	23,286..23,486	−	66	2 TM segments containing protein
28	23,599..24,228	−	209	2 Transmembrane segments containing protein
29	24,708..25,880	+	390	Tyrosine recombinase/integrase
30	26,063..25,845	−	72	MerR family regulatory protein
31	26,060..26,290	−	76	Hypothetical protein
32	26,287..26,538	−	83	Hypothetical protein
33	26,535..26,804	−	89	Hypothetical protein
34	26,963..27,997	−	344	Ead/Ea22-like protein
35	27,994..30,309	−	771	ParB partition protein family
36	30,325..30,552	−	75	Hypothetical protein
37	30,549..31,253	−	234	Deoxynucleoside monophosphate kinase
38	31,250..31,546	−	98	AAA+-type ATPase
39	31,556..31,936	−	126	ssDNA-binding protein
40	31,936..32,121	−	61	TM segment containing protein
41	32,118..32,252	−	44	Hypothetical protein
42	32,249..32,623	−	124	Hypothetical protein
43	32,620..32,859	−	79	Hypothetical protein
44	33,006..33,674	−	222	Repressor protein CI, S24 family peptidase
45	33,751..34,062	+	103	DNA-binding transcriptional regulator Cro-like
46	34,140..34,523	+	127	CII-like protein, XRE-type HTH domain
47	34,520..34,822	+	100	Hypothetical protein
48	34,822..37,485	+	887	Toprim domain containing protein
49	37,836..38,153	+	105	Hypothetical protein
50	38,140..38,544	+	134	Antiterminator Q protein
51	38,591..38,776	+	61	Hypothetical protein
52	38,939..39,298	+	119	HNH endonuclease

**Table 2:** Characteristics of Variovorax prophages identified in the genomic sequences from the NCBI database.

No.	Prophage	Strain (Accession No)	Coordinates	Virfam	Site of Integration	*att* Sequence	Type of Integrase	Genome Size (bp)
1.	PMC12 _pp_2	*Variovorax* sp. PMC12 (CP027773)	5,166,871.. 5,204,531	Siphoviridae of Type 1	tRNA-Ser(TGA)	TCTCACACTCTCCGCCAGAATCAATC(T/C)(T/C)TGGCAGTTTTTGAAA-GTCCCGCGCAGCCTCTGCGA	Tyrosine	37,661
2.	VAI-C_pp_1	*V. paradoxus* VAI-C (CP063166)	5,357,355.. 5,405,518	Siphoviridae of Type 1	tRNA-Ser(CGA)	TCCCTCCCTCTCCTCCAA	Tyrosine	48,164
3.	B4_pp_1	*V. paradoxus* B4 (CP003911)	4,696,119.. 4,740,974	Siphoviridae of Type 1	tRNA-Ser(TGA)	CACACTCTCCGCCAGAATCCATCTTTGGCAGTTTCTTGAAGTCCCGCGCAGTTCATGCGACGGGACTTTTTCATTGGG	Tyrosine	44,856
4.	CSUSB_pp_1	*V. paradoxus* CSUSB (CP046622)	3,365,369.. 3,408,809	Siphoviridae of Type 1	tRNA-Ser(GCT)	CCTCCGGTTCCGCCAA	Tyrosine	43,441
5.	J1_pp_1	*V. boronicumulans* J1 (CP023284)	2,675,825.. 2,721,941	Siphoviridae of Type 1	tRNA-Val(TAC)	CCCTTACAAGGCGTAGGTCGGGGGTTCGAGCCCCTCAGCACCCACCACCA	Tyrosine	46,117
6.	J1_pp_4	*V. boronicumulans* J1 (CP023284)	4,415,582.. 4,454,484	Siphoviridae of Type 1	tRNA dihydrouridine synthase DusA	GCGCTCGCTCGGG	Tyrosine	38,903
7.	vvax_pp_1	*V. paradoxus* vvax (LR743507)	4,988,968.. 5,029,379	Siphoviridae of Type 1	tRNA-Ser(TGA)	CTCGCGCAACCA	Tyrosine	40,412
8.	PAMC 26660_pp_1	*Variovorax* sp. PAMC 26660 (CP060295)	1,178,078.. 1,222,407	Siphoviridae of Type 1	Sigma-70 family RNA polymerase	GTTGCCCAGCTTCTTGCGCAGCCACGACTGGAGCCAGCCGTGGTGGTCGC	Transposase Mu-like	44,330
9.	RKNM96_pp_1	*Variovorax* sp. RKNM96 (CP046508)	6,243,874.. 6,284,473	Siphoviridae of Type 1	Intergenic region	TG…CA	Transposase Mu-like	40,600
10.	HW608_pp_3	*Variovorax* sp. HW608 (LT607803)	7,244,519.. 7,304,853	Siphoviridae of Type 1	Intergenic region	Not identified	Serine	60,335
11.	PAMC 28711_pp_2	*Variovorax* sp. PAMC 28711 (CP014517)	234,890.. 279,029	Siphoviridae of Type 1	Flavin reductase	ATGGACATCGACTTCGCCACCCTCACCGAATACCAGCGCTACAA	Tyrosine	44,140
12.	VAI-C_pp_2	*V. paradoxus* VAI-C (CP063166)	4,386,549.. 4,428,859	Siphoviridae of Type 1	DNA competence protein ComEC/Rec2	GCTGCCGTGGTGCGGC	Tyrosine	42,311
13.	J1_pp_2	*V. boronicumulans* J1 (CP023284)	3491486.. 3,531,295	Siphoviridae of Type 1	30S ribosomal S12 methylthiotransferase RimO	GTCGCCGGTCTTGGCG	Serine	39,810
14.	J1_pp_3	*V. boronicumulans* J1 (CP023284)	4,205,701.. 4,263,544	Siphoviridae of Type 1	tRNA-Arg(TCT)	ATCCCCTCCGG	Tyrosine	52,010
	VarioGold	Variovorax sp. ZS18.2.2	-	Siphoviridae of Type 1	tRNA-Ser(CGA)	CCTCCCTCTCCTCCA	Tyrosine	39,429
15.	5C-2_pp_1	*V. paradoxus* 5C-2 (CP045644)	3,926,575.. 4,039,667	Siphoviridae of Type 1	tRNA-Pro(GGG)	TTGCATGGGGTGCAAGGGGTCGAAGGTTCGAATCCTTTCACACCG-ACCAATAA	Tyrosine	113,093
16.	PBS-H4_pp_2	*Variovorax* sp. PBS-H4 (LR594675)	3,082,071.. 3,170,589	Siphoviridae of Type 1	tRNA-Gly(CCC)	GTTCTACCATTGAACTACACCCGCA	Tyrosine	88,469
17.	HW608_pp_2	*Variovorax* sp. HW608 (LT607803)	5,030,346.. 5,096,253	Siphoviridae of Type1	peptidylprolyl isomerase	TCCATACGAGAATTC-TCC	Tyrosine	60,846
18.	PMC12_pp_3	*Variovorax* sp. PMC12 (CP027773)	5,692,545.. 5,758,265	Podoviridae of Type 3	Intergenic region	CTGGCTACCCG(C/G)CT(A/G)GCTACCC	Tyrosine	65,721
19.	PDNC026_pp_1	*Variovorax* sp. PDNC026 (CP070343)	5,173,628.. 5,241,433	Podoviridae of Type 3	tRNA-His(GTG)	CAGATTGTGATTCTGGTCGTCGTGGGTTCGAGTCCCATCAGCCACCCCAA	Tyrosine	67,886
20.	PMC12 _pp_4	*Variovorax* sp. PMC12 (CP027773)	2,605,254.. 2,666,437	Podoviridae of Type 3	tRNA-Leu(CAA)	TGTGGTGCCCGGGGCCGGAATCGAACCGGCACACCTTTCGGTGGGGGATTTTGAGTCCC	Tyrosine	61,184
21.	EPS_pp_1	*V. paradoxus* EPS (CP002417)	2,169,141.. 2,234,652	Podoviridae of Type 3	tRNA-His(GTG)	CAGATTGTGATTCTGGTCGTCGTGGGTTCGAGTCCCATCAGCCACCCCAA	Tyrosine	65,512
22.	VAI-C_pp_3	*V. paradoxus* VAI-C CP063166	2,942,097.. 3,007,349	Podoviridae of Type 3	tRNA-Asn(GTT)	TGGCTCCTCGACCTGGGCTCGAACCAGGGA-CCTACGGATTAACAGTC	Tyrosine	65,253
23.	38R_pp_1	*Variovorax* sp. 38R (CP062121)	3,617,751.. 3,688,219	Podoviridae of Type 3	tRNA-Arg(TCT)	Not identified	Tyrosine	70,469
24.	PAMC 28711 _pp_1	*Variovorax* sp. PAMC 28711 (CP014517)	42,947.. 81,571	Podoviridae of Type 3	tRNA-Arg(TCT)	TGGCCTGTCCGGAGGGGATCGAACCCCCGACAACCTGCTTAGAAGGCAG	Tyrosine	38,625
25.	RA8_pp_1	*Variovorax* sp. RA8 (LR594662)	5,162,402.. 5,202,563	Podoviridae of Type 3	tRNA-Arg(ACG)	GGCTACGAACCAAGGGGTCGTGGGTTCGAATCCTGCCAGCCGCACCACTTTT	Tyrosine	40,162
26.	PBL-E5_pp_1	*Variovorax* sp. PBL-E5 (LR594671)	4,567,056.. 4,606,930	Podoviridae of Type 3	tRNA-Arg(CCT)	TGGTGCCCTCGACAGGAATCGAACCTG	Tyrosine	39,875
27.	WDL1_pp_1	*Variovorax* sp. WDL1 (LR594689)	2,078,592.. 2,121,776	Podoviridae of Type 3	tRNA-Arg(CCT)	AGGTTCGATTCCTGTCGAGGGCACCAGTAAGGT	Tyrosine	43,185
28.	PBS-H4_pp_3	*Variovorax* sp. PBS-H4 (LR594675)	4,606,795.. 4,670,479	Podoviridae of Type 3	tRNA-His(GTG)	CAGATTGTGATTCTGGTCGTCGTGGGTTCGAGTCCCATCAGCCACCCCAA	Tyrosine	63,685
29.	PAMC 26660_pp_2	*Variovorax* sp. PAMC 26660 (CP060295)	4,885,415.. 4,937,289	Podoviridae of Type 3	tRNA-His(GTG)	CAGATTGTGATTCTGGTCGTCGTGGGTTCGAGTCCCATCAGCCACCCCAA	Tyrosine	51,952
30.	VAI-C_pp_4	*V. paradoxus* VAI-C (CP063166)	3,837,980.. 3,880,010	Podoviridae of Type 3	tRNA-Ser(GCT)	TTGGCGGAACCGGAGG	Tyrosine	42,031
31.	PMC12_pp_1	*Variovorax* sp. PMC12 (CP027773)	1,531,523.. 1,574,372	Podoviridae of Type 3	tRNA-Leu(TAA)	TTCGGGGCACCA	Tyrosine	42,264
32.	RKNM96_pp_2	*Variovorax* sp. RKNM96 (CP046508)	4,013,608.. 4,057,349	Podoviridae of Type 3	tRNA-Asn(GTT)	ACTGTTAATCCGTAGGTCCCTGGTTCGAGCCCAGGTCGAGGAGCCA	Tyrosine	43,742
33.	PBS-H4_pp_1	*Variovorax* sp. PBS-H4 (LR594675)	1,630,279.. 1,670,591	Podoviridae of Type 3	tRNA-Ser(CGA)	TCCCACCCTCTCCGCCAGCA	Tyrosine	40,313
34.	PAMC 26660_pp_3	*Variovorax* sp. PAMC 26660 (CP060295)	4,563,808.. 4,646,346	Myoviridae of Type 1	Intergenic region	CGGGGGTTCAAATCCCCCCA	Tyrosine	82,539
35.	WDL1_pp_2	*Variovorax* sp. WDL1 (LR594689)	662,952.. 719,595	Myoviridae of Type 1	tRNA-Ser(ACT)	GTAGTGGCTCCTCGACCTGGGCTCGAACCAGGGACCTACGGATTAACAG	Tyrosine	56,644
36.	J1_pp_5	*V. boronicumulans* J1 (CP023284)	6,754,149.. 6,793,076	Myoviridae of Type 1	tRNA-Met(CAT)	TGGTTGCGCGAG	Tyrosine	38,928
37.	PDNC026_pp_2	*Variovorax* sp. PDNC026 (CP070343)	4,474,061.. 4,514,959	Myoviridae of Type 1	tRNA-Arg(TCT)	TTGGCCTGCCCGGAGGGGATCGAACC	Serine	40,899

## Supplementary Materials

The following supporting information can be downloaded at: https://www.mdpi.com/article/10.3390/ijms232113539/s1. References [93,94,95,96] are cited in the supplementary materials.
