# Supplementary material for: Characteristics and Comparative Genomic Analysis of a Novel Virus, VarioGold, the First Bacteriophage of Variovorax
Source: Int J Mol Sci. 2022 Nov 4;23(21):13539. doi: 10.3390/ijms232113539 (PMC9658991; doi:10.3390/ijms232113539)
Supplement: Supplementary file 1 [file ijms-23-13539-s001.zip › ijms-1984362-supplementary.pdf]

Supplementary materials for

Characteristics and comparative genomic analysis of a novel virus, VarioGold, the first bacteriophage of *Variovorax*

Przemyslaw Decewicz, Michal Kitowicz and Monika Radlinska *

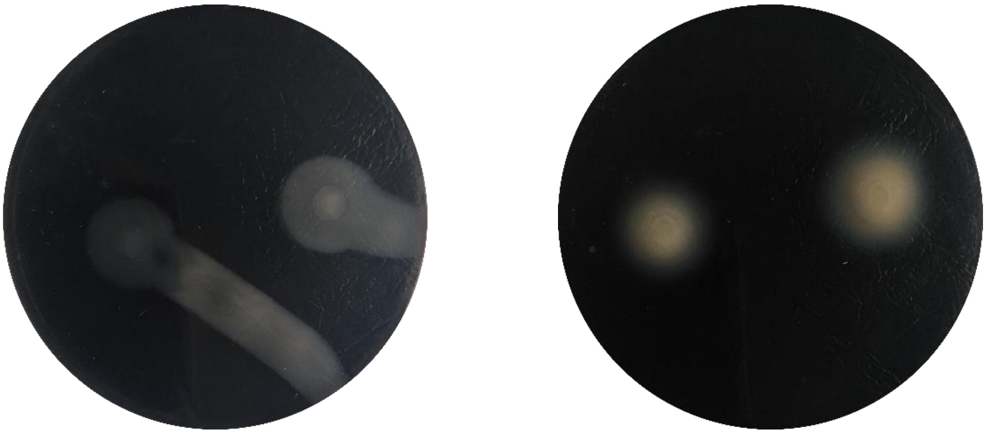

(**a**) (**b**)

Figure S1. Surface motility of *Variovorax* sp. ZS18.2.2 similar to swarming motion on: (a) R2A; (b) LB semi-solids. The photograph was taken after 72h of incubation at 20°C. 10 µl of *Variovorax* sp. ZS18.2.2 overnight culture was applied at two spots on each plate with 0.5% R2A/LB semi-solid media. An analogous result was observed after similar time of incubation at 4 and 10°C (on both media) and at 30°C (only on the R2A).

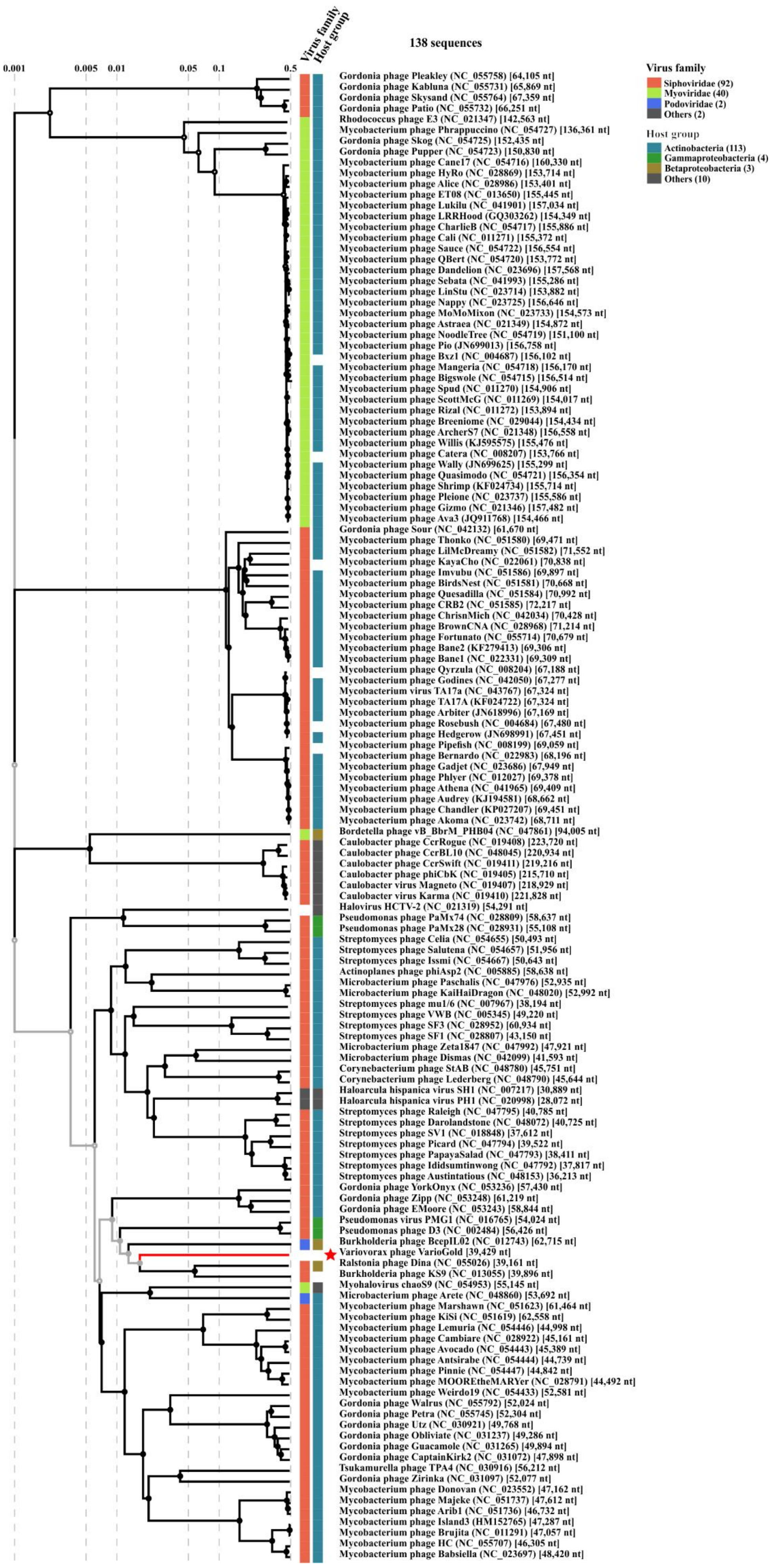

Figure S2. Proteomic-based tree of VarioGold and dsDNA viruses represented in the rectangular view generated with ViPTree software.

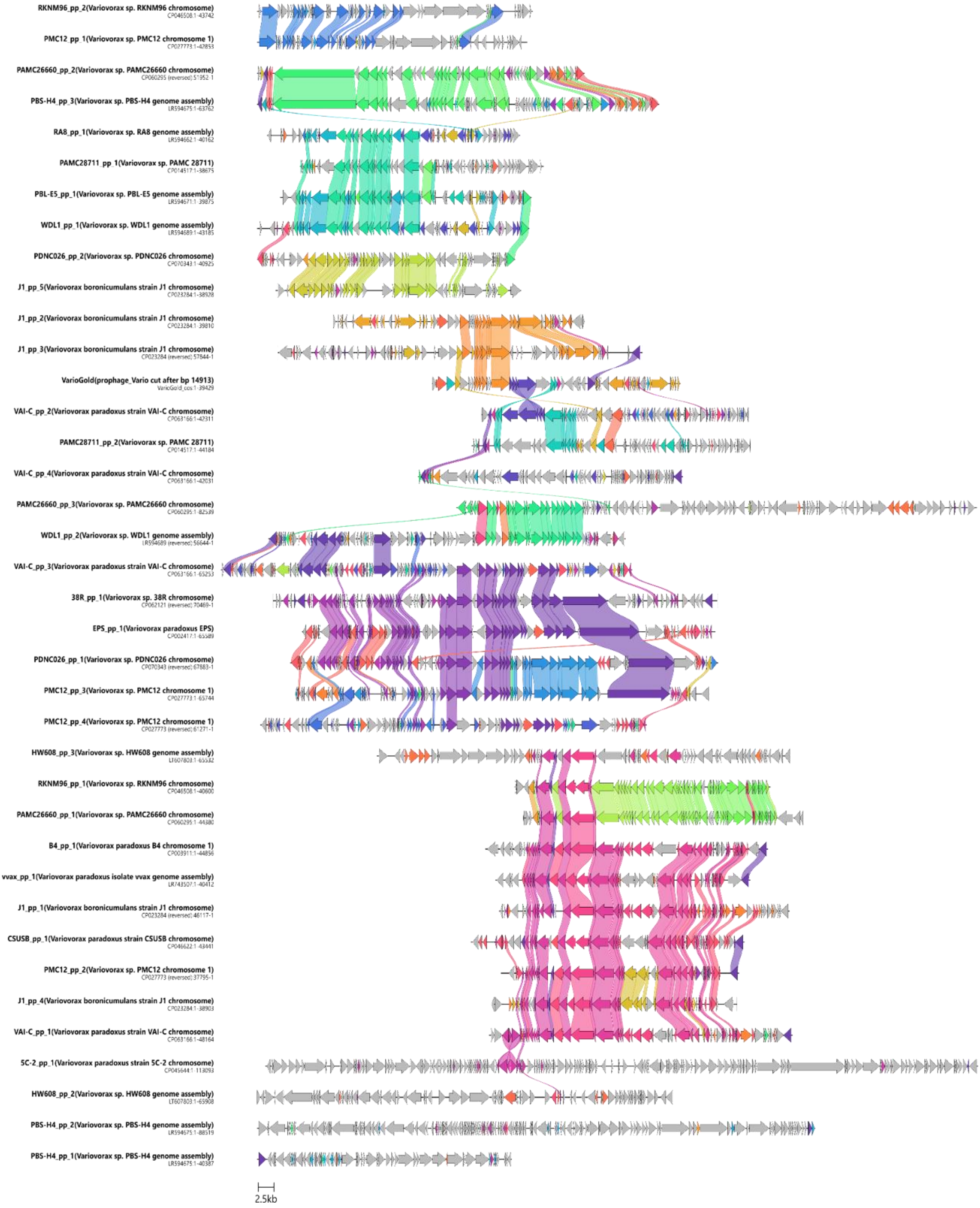

Figure S3. The genome alignment of the *Variovorax* (pro)phages. Phage genomes were delineated and reversed if required. Each protein-encoding feature was presented as an arrow. The colour of the arrow reflects a group of at least two proteins with a minimum 30 % sequence identity. These were additionally connected with blocks of the same colour.

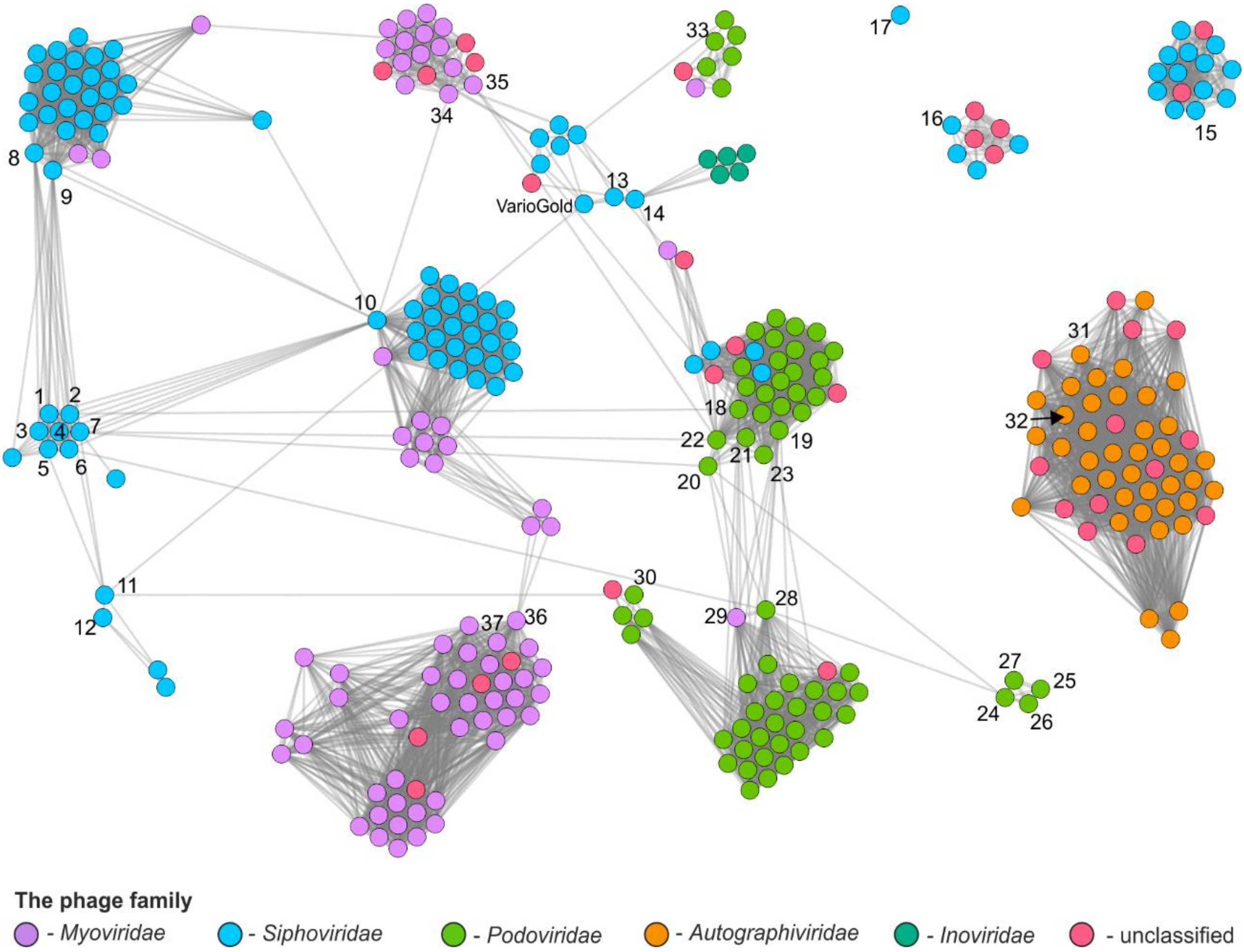

Figure S4. Proteome-based similarity network. *Variovorax* (pro)phages identified by us and viruses reflecting significant similarity to them were shown as nodes. The similarity is reflected by the presence of an edge between particular nodes. The colouring (in line with the key) refers to the previous taxonomic classification of *Caudoviricetes* families. Numbers next to the nodes symbolising *Variovorax* prophages correspond to the numbering in Table 2.

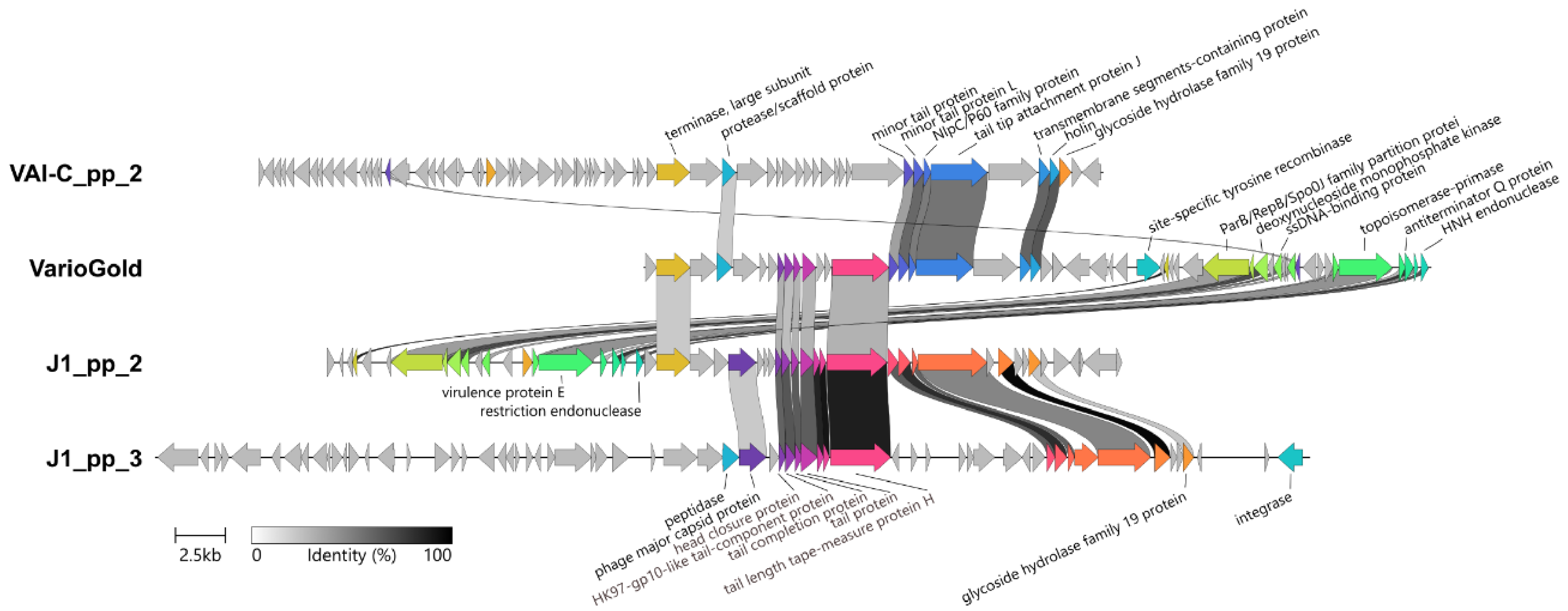

Figure S5. The genome alignment of the VarioGold, J1_pp_2, J1_pp_3 and VAI-C_pp_2 (pro)phages. Phage genomes were delineated and reversed if required. Each protein-encoding feature was presented as an arrow. The colour of arrow reflects a group of least two proteins with at least 30 % sequence identity. The colour of blocks connecting each arrow pair represents the level of sequence identity according to white-to-black scale. The arrows with products annotated as hypothetical proteins were not labelled.

Table S1. List of *Variovorax* strains characterized in terms of resistance to As (III) and As(V).

**Strain ID**	**Sample type**	**Country/Territory of origin**	**MTC* [mM]**		**Reference**
			**As (III)**	**As(V)**	
Variovorax sp. MM-1	Contaminated soil near Lead Smelter Plant	Australia	20	200	[1]
Variovorax sp. S3	Woodland soil	USA	0	30	[2]
Variovorax sp. S12	Woodland soil	USA	0	150	[2]
V. paradoxus DhaiF	As-contaminated agronomic soil	Bangladesh	15	30	[3]
V. gossypii JM-310	Internal root tissue of upland cotton	USA	9	ND	[4,5]
V. paradoxus S110	Interior of a potato plant	USA	8,9	ND	[4,6]

* MTC – maximum tolerable concentration; ND – no data

Table S2. List of *Variovorax* strains used in prophage identification.

Strain ID	Accession No.	Genome size [bp]	Sample Type	Country/Territory of origin
V. paradoxus EPS	CP002417	6,550,056	Soil from the coastal sage scrub rhizosphere	USA
V. *paradoxus* B4	CP003911	7,148,516	Contaminated soil near a chemical factory	ND
Variovorax sp. PAMC28711	CP014517	4,316,152	Thallus of Antarctic lichen *Himantormia* sp.	Antarctica
V. boronicumulans J1	CP023284	7,137,898	Unspecified soil	China
Variovorax sp. PMC12	CP027773	7,015,237	Potting soil from tomato rhizosphere	South Korea
V. paradoxus 5C-2	CP045644	7,288,087	Heavy metals-contaminated mining waste	Italy
V. paradoxus CSUSB	CP046622	5,574,400	Lower chaparral/coastal sage brush soil from sunflower	USA
Variovorax sp. PAMC26660	CP060295	7,388,698	Antarctic lichen	Antarctica
Variovorax sp. PAMC28562	CP060296	4,693,528	Unspecified soil	Antarctica
Variovorax sp. 38R	CP062121	6,870,625	Atrazine-contaminated agronomic soil	France
Variovorax sp. PDNC026	CP070343	7,110,136	Plastic debris in land/lake environment	USA
Variovorax sp. RKNM96	CP046508	7,168,845	Unspecified soil	Canada
Variovorax sp. HW608	LT607803	7,733,665	Soil of the Luqillo Experimental Forest	Puerto Rico
Variovorax sp. PBL-H6	LR594659	6,871,554	Linurion-contaminated agronomic soil	Belgium
Variovorax sp. SRS16	LR594666	7,674,934	Linurion-contaminated agronomic soil	Denmark
Variovorax sp. RA8	LR594662	7,424,241	Riverbed sediment	Japan
Variovorax sp. PBL-E5	LR594671	7,086,410	Linurion-contaminated agronomic soil	Denmark
Variovorax sp. PBS-H4	LR594675	6,652,076	Linurion-contaminated agronomic soil	Belgium
Variovorax sp. WDL1	LR594689	8,360,137	Linurion-contaminated agronomic soil	Belgium
V. paradoxus VAI-C	CP063166	9,439,028	College campus soil	USA
V. paradoxus vvax (LAD61)	LR743507	7,257,405	The leaf of common dandelion	ND

ND – no data

References

1. Bahar, M.M.; Megharaj, M.; Naidu, R. Kinetics of arsenite oxidation by Variovorax sp. MM-1 isolated from a soil and identification of arsenite oxidase gene. *J Hazard Mater* **2013**, *262*, 997-1003, doi:10.1016/j.jhazmat.2012.11.064.
2. Jackson, C.R.; Harrison, K.G.; Dugas, S.L. Enumeration and characterization of culturable arsenate resistant bacteria in a large estuary. *Syst Appl Microbiol* **2005**, *28*, 727-734, doi:10.1016/j.syapm.2005.05.012.
3. Bachate, S.P.; Cavalca, L.; Andreoni, V. Arsenic-resistant bacteria isolated from agricultural soils of Bangladesh and characterization of arsenate-reducing strains. *J Appl Microbiol* **2009**, *107*, 145-156, doi:10.1111/j.1365-2672.2009.04188.x.
4. Flores-Duarte, N.J.; Pérez-Pérez, J.; Navarro-Torre, S.; Mateos-Naranjo, E.; Redondo-Gómez, S.; Pajuelo, E.; Rodríguez-Llorente, I.D. Improved. *Plants (Basel)* **2022**, *11*, doi:10.3390/plants11081091.
5. Kämpfer, P.; Busse, H.J.; McInroy, J.A.; Glaeser, S.P. Variovorax gossypii sp. nov., isolated from Gossypium hirsutum. *Int J Syst Evol Microbiol* **2015**, *65*, 4335-4340, doi:10.1099/ijsem.0.000581.
6. Han, J.I.; Choi, H.K.; Lee, S.W.; Orwin, P.M.; Kim, J.; Laroe, S.L.; Kim, T.G.; O'Neil, J.; Leadbetter, J.R.; Lee, S.Y.; et al. Complete genome sequence of the metabolically versatile plant growth-promoting endophyte Variovorax paradoxus S110. *J Bacteriol* **2011**, *193*, 1183-1190, doi:10.1128/JB.00925-10.
